# Prediction of Pathological Stage in Patients with Prostate Cancer: A Neuro-Fuzzy Model

**DOI:** 10.1371/journal.pone.0155856

**Published:** 2016-06-03

**Authors:** Georgina Cosma, Giovanni Acampora, David Brown, Robert C. Rees, Masood Khan, A. Graham Pockley

**Affiliations:** 1 Computing and Technology, School of Science and Technology, Nottingham Trent University, Nottingham, United Kingdom; 2 John van Geest Cancer Research Centre, Nottingham Trent University, Nottingham, United Kingdom; 3 Department of Urology, University Hospitals of Leicester NHS Trust, Leicester, United Kingdom; Southern Illinois University School of Medicine, UNITED STATES

## Abstract

The prediction of cancer staging in prostate cancer is a process for estimating the likelihood that the cancer has spread before treatment is given to the patient. Although important for determining the most suitable treatment and optimal management strategy for patients, staging continues to present significant challenges to clinicians. Clinical test results such as the pre-treatment Prostate-Specific Antigen (PSA) level, the biopsy most common tumor pattern (Primary Gleason pattern) and the second most common tumor pattern (Secondary Gleason pattern) in tissue biopsies, and the clinical T stage can be used by clinicians to predict the pathological stage of cancer. However, not every patient will return abnormal results in all tests. This significantly influences the capacity to effectively predict the stage of prostate cancer. Herein we have developed a neuro-fuzzy computational intelligence model for classifying and predicting the likelihood of a patient having Organ-Confined Disease (OCD) or Extra-Prostatic Disease (ED) using a prostate cancer patient dataset obtained from The Cancer Genome Atlas (TCGA) Research Network. The system input consisted of the following variables: Primary and Secondary Gleason biopsy patterns, PSA levels, age at diagnosis, and clinical T stage. The performance of the neuro-fuzzy system was compared to other computational intelligence based approaches, namely the Artificial Neural Network, Fuzzy C-Means, Support Vector Machine, the Naive Bayes classifiers, and also the AJCC pTNM Staging Nomogram which is commonly used by clinicians. A comparison of the optimal Receiver Operating Characteristic (ROC) points that were identified using these approaches, revealed that the neuro-fuzzy system, at its optimal point, returns the largest Area Under the ROC Curve (AUC), with a low number of false positives (FPR = 0.274, TPR = 0.789, AUC = 0.812). The proposed approach is also an improvement over the AJCC pTNM Staging Nomogram (*FPR* = 0.032, *TPR* = 0.197, *AUC* = 0.582).

## Introduction

Cancer staging prediction is a process for estimating the likelihood that the disease has spread before treatment is given to the patient. Cancer staging evaluation occurs before (i.e. at the prognosis stage) and after (i.e. at the diagnosis stage) the tumor is removed—the clinical and pathological stages respectively. The clinical stage evaluation is based on data gathered from clinical tests that are available prior to treatment or the surgical removal of the tumor. There are three primary clinical stage tests for prostate cancer: the Prostate Specific Antigen (PSA) test which measures the level of PSA in the bloodstream; a biopsy which is used to detect the presence of cancer in the prostate and to evaluate the degree of cancer aggressiveness (results are usually given in the form of the Primary and Secondary Gleason patterns); and a physical examination, namely the Digital Rectal Examination (DRE) which can determine the existence of disease and possibly provide sufficient information to predict the stage of the cancer. A limitation of the PSA test is that abnormally high PSA levels may not necessarily indicate the presence of prostate cancer, nor might normal PSA levels reflect the absence of prostate cancer. Pathological staging can be determined following surgery and the examination of the removed tumor tissue, and is likely to be more accurate than clinical staging, as it allows a direct insight into the extent and nature of the disease. More information on the clinical tests is provided in the next subsection *Medical Background*.

Given the potential prognostic power of the clinical tests, a variety of prostate cancer staging prediction systems have been developed. The ability to predict the pathological stage of a patient with prostate cancer is important, as it enables clinicians to better determine the optimal treatment and management strategies. This is to the patient’s considerable benefit, as many of the therapeutic options can be associated with significant short- and long- term side-effects. For example, radical prostatectomy (RP)—the surgical removal of the prostate gland—offers the best chance for curing the disease when prostate cancer is localised, and the accurate prediction of pathological stage is fundamental to determining which patients would benefit most from this approach [1–3]. Currently, clinicians use nomograms to predict a prognostic clinical outcome for prostate cancer, and these are based on statistical methods such as logistic regression [4]. However, cancer staging continues to present significant challenges to the clinical community.

The prostate cancer staging nomograms which are used to predict the pathological stage of the cancer are based on results from the clinical tests. However, the accuracy of the nomograms is debatable [5, 6]. Briganti et al. [5] argues that nomograms are accurate tools and that “Personalized medicine recognizes the need for adjustments, according to disease and host characteristics. It is time to embrace the same attitude in other disciplines of medicine. This includes urologic oncology where nomograms, regression-trees, lookup tables and neural networks represent the key tools capable of providing individualized predictions”. Dr Joniau in [5] argues that the data used for devising the nomograms are subjective and, to a certain extent, biased by institutional protocols on which patients are selected for a given treatment. Dr Joniau states that one of the drawbacks of nomograms is that various nomograms have been devised for risk estimation and it is difficult to determine which nomogram will provide the most reliable risk estimation for a particular patient. He emphasises that although nomograms allow for more accurate risk assessment, this risk estimation is a “snapshot in a risk continuum”. Although this might allow personalized predictions, it also makes treatment decisions difficult [5].

Cancer prediction systems which consider various variables for the prediction of an outcome require computational intelligent methods for efficient prediction outcomes [7]. Although computational intelligence approaches have been used to predict prostate cancer outcomes, very few models for predicting the pathological stage of prostate cancer exist. In essence, classification models based on computational intelligence are utilised for prediction tasks. Classification is a form of data analysis which extracts classifier models describing data classes, and uses these models to predict categorical labels (classes) or numeric values [8]. When the classifier is used to predict a numeric value, as opposed to a class label, it is referred to as a predictor. Classification and numeric prediction are both types of prediction problems [8], and classification models are widely adopted to analyse patient data and extract a prediction model in the medical setting.

Computational intelligence approaches, and in particular fuzzy-based approaches, are based on mathematical models that are specially developed for dealing with the uncertainty and imprecision which is typically found in the clinical data that are used for prognosis and the diagnosis of diseases in patients. These characteristics make these algorithms a suitable platform on which to base new strategies for diagnosing and staging prostate cancer. For example, not everyone diagnosed with prostate cancer will exhibit abnormal results in all tests, as a consequence of which, different test result combinations can lead to the same outcome.

The capacity of fuzzy, and especially neuro-fuzzy approaches, to predict the pathological stage of prostate cancer has not been as widely evaluated as the more commonly used Artificial Neural Network (ANN) and other approaches. However, fuzzy approaches have been applied to other prostate cancer scenarios. Benechi et al. [9] have applied the Co-Active Neuro-Fuzzy Inference System (CANFIS) to predict the presence of prostate cancer; Keles et al.[10] proposed a neuro-fuzzy system for predicting whether an individual has cancer or Benign Prostatic Hyperplasia (BPH, a benign enlargement of the prostate). Çinar [11] designed a classifier-based expert system for the early diagnosis of prostate cancer, thereby aiding the decision-making process and informing the need for a biopsy. Castanho et al. [12] developed a genetic-fuzzy expert system which combines pre-operative serum PSA, clinical stage, and Gleason grade of a biopsy to predict the pathological stage of prostate cancer (i.e. whether it was confined or not-confined).

Saritas et al. [13] devised an ANN approach for the prognosis of cancer which can be used to assist clinical decisions relating to the necessity for a biopsy. Shariat et al. [14] have performed a critical review of prostate cancer prediction tools and concluded that predictive tools can help during the complex decision-making processes, and that they can provide individualised, evidence-based estimates of disease status in patients with prostate cancer.

Finally, Tsao et al. [15] developed an ANN model to predict prostate cancer pathological staging in 299 patients prior to radical prostatectomy, and found that the ANN model was superior at predicting Organ Confined Disease in prostate cancer than a Logistic Regression model. Tsao et al. [15] also compared their ANN model with Partin Tables, and found that the ANN model more accurately predicted the pathological stage of prostate cancer.

Herein we propose a neuro-fuzzy model for predicting the pathological stage of prostate cancer. The system inputs comprise the following variables: the most common tumor pattern (Primary Gleason pattern), the second most common pattern (Secondary Gleason pattern), PSA levels, age at diagnosis, and clinical T stage. The neuro-fuzzy model automatically constructs fuzzy rules via a training process which is applied to existing and known patient records and status. These rules are then used to predict the prostate cancer stage of patients in a validation set. The model makes use of the Adaptive Neuro-Fuzzy Inference System which is also used to optimise the predictive performance. The outcome for each patient record is a numerical prediction of the ‘degree of belongingness’ of each patient in the Organ-Confined Disease and Extra-Prostatic Disease classes.

### Medical Background

This section describes the variables used for diagnosis.

#### Prostate Specific Antigen (PSA)

The Prostate Specific Antigen (PSA) test is a blood test that measures the level of prostate-specific antigen in the bloodstream. Although having limitations, the PSA test is currently the best method for identifying an increased risk of localised prostate cancer. PSA values tend to rise with age, and the total PSA levels (ng/ml) recommended by the Prostate Cancer Risk Management Programme are as follows [16]: 50–59 years, *PSA* ≥ 3.0; 60–69 years, *PSA* ≥ 4.0; and 70 and over, *PSA* > 5.0. Abnormally high and raised PSA levels may, but does not necessarily, indicate the presence of prostate cancer. The European Study of Screening for Prostate Cancer revealed that screening significantly reduces death from prostate cancer, and that a man who undergoes PSA testing will have his risk of dying from prostate cancer reduced by 29% [17, 18], and [19]. However, it should also be noted that a normal PSA test does not necessarily exclude the presence of prostate cancer.

#### Primary and Secondary Gleason Patterns

A tissue sample (biopsy) is used to detect the presence of cancer in the prostate and to evaluate its aggressiveness. The results from a prostate biopsy are usually provided in the form of the Gleason grade score. For each biopsy sample, pathologists examine the most common tumor pattern (Primary Gleason pattern) and the second most common pattern (Secondary Gleason pattern), with each pattern being given a grade of 3 to 5. These grades are then combined to create the Gleason score (a number ranging from 6 to 10) which is used to describe how abnormal the glandular architecture appears under a microscope. For example, if the most common tumor pattern is grade 3, and the next most common tumor pattern is grade 4, the Gleason score is 3 + 4, or 7. A score of 6 is regarded as low risk disease, as it poses little danger of becoming aggressive; and a score of 3 + 4 = 7 indicates intermediate risk. Because the first number represents the majority of abnormal tissue in the biopsy sample, a 3 + 4 is considered less aggressive than a 4 + 3. Scores of 4 + 3 = 7, or 8 to 10 indicate that the glandular architecture is increasingly more abnormal and associated with high risk disease which is likely to be aggressive.

#### Clinical and Pathological Stages

The clinical stage is an estimate of the prostate cancer stage, and this is based on the results of the digital rectal examination (DRE). The pathological stage can be determined if a patient has had surgery and hence is based on the examination of the removed tissue. Pathological staging is likely to be more accurate than clinical staging, as it can provide a direct insight into the extent of the disease. At the clinical stage, there are four categories for describing the local extent of a prostate tumor (T1 to T4). Clinical and pathological staging use the same categories, except that the T1 category is not used for pathological staging. In summary, stages T1 and T2 describe a cancer that is probably organ-confined, T3 describes cancer which is beginning to spread outside the prostate, and T4 describes a cancer that has likely begun to spread to nearby organs. Category T1 is when the tumor cannot be felt during the DRE or be seen with imaging such as transrectal ultrasound (TRUS). Category T1 has three subcategories: T1a cancer is found incidentally during a transurethral resection of the prostate (TURP) which will have been performed for the treatment of Benign Prostatic Hyperplasia, and the cancer is present in no more than 5% of the tissue removed; T1b cancer is found during a TURP, but is present in more than 5% of the tissue removed, and T1c cancer is found in a needle biopsy which has been performed due to an elevated PSA level. Category T2 is when the tumor can be felt during a DRE or seen with imaging, but still appears to be confined to the prostate gland. Category T2 has three subcategories: T2a cancer is in one half or less of only one side (left or right) of the prostate; T2b cancer is in more than half of only one side (left or right) of the prostate; and T2c cancer is in both sides of the prostate. Category T3 has two subcategories: T3a cancer extends outside the prostate, but not to the seminal vesicles; and T3b cancer has spread to the seminal vesicles. Finally, category T4 cancer has grown into tissues next to the prostate (other than the seminal vesicles), such as the urethral sphincter, the rectum, the bladder, and/or the wall of the pelvis.

The TNM staging is the most widely used system for prostate cancer staging and aims to determine the extent of:

- primary tumor (T stage),
- the absence or presence of regional lymph node involvement (N stage), and
- the absence or presence of distant metastases (M stage)

The TNM system has been accepted by the Union for International Cancer Control (UICC) and the American Joint Committee on Cancer (AJCC). Most medical facilities use the TNM system as their main method for cancer reporting. The clinical TNM and pathological TNM are provided in Tables 1 and 2 respectively. Once the T, N, and M are determined, a stage of I, II, III, or IV is assigned, with stage I being early and stage IV being advanced disease. Upon determining the T, N, and M stages, a prognosis can be made about the anatomic stage of cancer using the groupings shown in Table 3 where a stage of I, II, III, or IV is assigned to a patient, with stage I being early and stage IV being advanced disease [20]. Stages I, II, are organ confined cancer stages, whereas Stages III and IV are extra-prostatic stages. TNM systems have gone through several refinements in order to “improve the uniformity of patient evaluation and to maintain a clinically relevant evaluation” [20]. In the most recent American Joint Committee on Cancer (AJCC) [21], the Gleason score and PSA have been incorporated in the cancer stage/prognostic groups 3.

**Table 1:** Definitions of clinical TNM according AJCC 2010 [21].

**Primary tumor (pT)**	
TX	Primary tumor cannot be assessed
T0	No evidence of primary tumor
**Clinically inapparent tumor neither palpable nor visible by imaging (T1)**	
T1a	Tumor incidental histologic finding in ≤ 5% of tissue resected
T1b	Tumor incidental histologic finding in > 5% of tissue resected
T1c	Tumor identified by needle biopsy (e.g. because of elevated PSA)
**Tumor confined within prostate (T2)**	
T2a	Tumor involves one-half of one lobe or less
T2b	Tumor involves more than one-half of one lobe but not both lobes
T2c	Tumor involves both lobes
**Tumor extends through the prostate capsule (T3)**	
T3a	Extracapsular extension (unilateral or bilateral)
T3b	Tumor invades seminal vesicle(s)
T4	Tumor is fixed or invades adjacent structures other than seminal vesicles such as external sphincter, rectum, bladder, levator muscles, and/or pelvic wall
**Regional lymph nodes (pN)**	
NX	Regional lymph nodes were not assessed
N0	No regional lymph node metastasis
N1	Metastasis in regional lymph node(s)
**Distant metastasis (pM)**	
M0	No distant metastasis
M1	Distant metastasis
M1a	Non-regional lymph node(s)
M1b	Bone(s)
M1c	Other site(s) with or without bone disease

**Table 2:** Pathological TNM according AJCC 2010 [21]. There is no pT1 classification.

**Organ confined (pT2)**	
pT2a	Unilateral, one-half of one side or less
pT2b	Unilateral, involving more than one-half of one side, but not both sides
pT2c	Bilateral disease
**Extraprostatic extension (pT3)**	
pT3a	Extraprostatic extension or microscopic bladder neck invasion
pT3b	Seminal vesicle invasion
pT4	Invasion of rectum levator muscles, and/or pelvic wall
**Regional lymph nodes (pN)**	
pNX	Regional lymph nodes not sampled
pN0	No positive regional lymph nodes
pN1	Metastasis in regional lymph node(s)
**Distant metastasis (pM)**	
pM1	Distant metastasis
pM1a	Non-regional lymph node(s)
pM1b	Bone(s)
pM1c	Other site(s) with or without bone disease

**Table 3:** Anatomic stage/prognostic groups (from AJCC 2010) [21].

Group	T	N	M	PSA	Gleason score (GS)
I	T1a–c	N0	M0	*PSA* < 10	*GS* ≤ 6
	T2a	N0	M0	*PSA* < 10	*GS* ≤ 6
	T1–2a	N0	M0	PSA X	GS X
IIA	T1a–c	N0	M0	*PSA* < 20	GS 7
	T1a–c	N0	M0	*PSA* ≥ 10 < 20	*GS* ≤ 6
	T2a	N0	M0	*PSA* < 20	*GS* ≤ 7
	T2b	N0	M0	*PSA* < 20	*GS* ≤ 7
	T2b	N0	M0	PSA X	GS X
IIB	T2c	N0	M0	Any PSA	Any GS
	T1–2	N0	M0	*PSA* ≥ 20	Any GS
	T1–2	N0	M0	Any PSA	*GS* ≥ 8
III	T3a–b	N0	M0	Any PSA	Any GS
IV	T4	N0	M0	Any PSA	Any GS
	Any T	N1	M0	Any PSA	Any GS
	Any T	Any N	M1	Any PSA	Any GS

## Methods I—Neuro-Fuzzy Model

Fuzzy logic is an extension of multivalued logic that deals with approximate, rather than fixed and exact reasoning. Fixed reasoning is the traditional binary logic where variables may take on true or false values. Fuzzy logic starts with the concept of a fuzzy set [22], which is a set without a crisp, clearly defined boundary. A fuzzy set can contain elements with only a partial degree of membership, and hence allows for degrees of truth, making fuzzy logic applicable to medical scenarios which are considered to involve complexity, uncertainty and vagueness. Fuzzy logic has been combined with various soft computing methodologies, including neuro-computing, thereby leading to powerful neuro-fuzzy systems.

**Fig 1:**
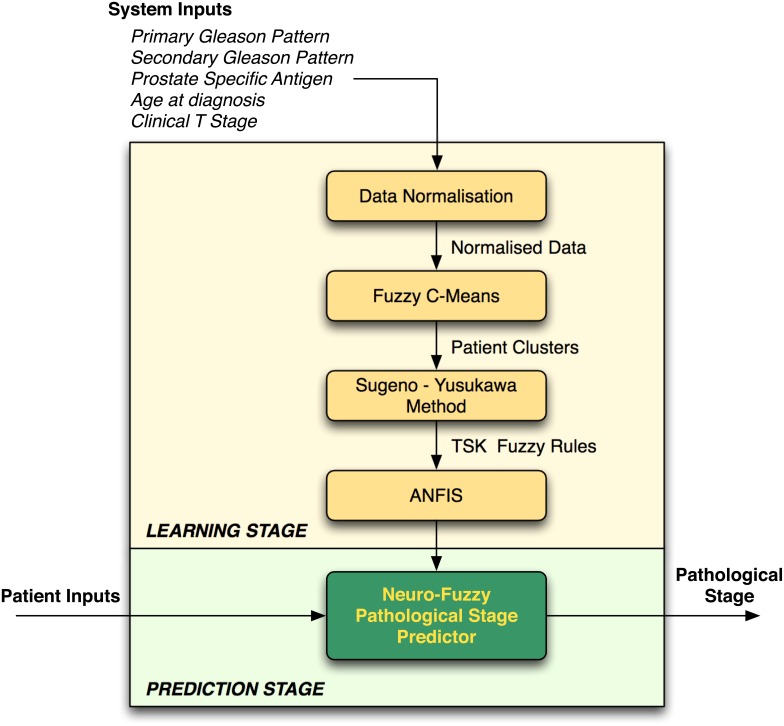
Neuro-Fuzzy Prostate Cancer Pathological Stage Predictor.

The neuro-fuzzy system proposed herein (a combination of fuzzy logic-based algorithms that are illustrated in Fig 1) predicts the pathological stage of cancer (i.e. diagnosis outcome), using data that are obtained from pre-operative clinical tests that are conducted at the prognosis stage. As with the TNM system, our proposed neuro-fuzzy system predicts whether a patient has organ-confined disease (OCD, pathological stage pT2) or extra-prostatic disease (ED, pathological stage > *pT*2).

The clinical data used for pathological cancer stage prediction are typically affected by imprecision, primarily due to the fact that not all patients exhibit abnormal results in all clinical tests. This poses a problem when trying to predict the progression of the cancer and therefore deciding on the best treatment strategy for patients. Hence, fuzzy logic is a suitable approach for this type of clinical prediction because it can be used to model human reasoning—in real scenarios the clinician would consider the data and give an estimation rather than a definite answer. The neuro-fuzzy system will make a prediction about a particular patient and return a value representing the ‘degree of membership’ of the patient’s cancer in the extra-prostatic set. The proposed framework is illustrated in Fig 1 and described in the subsections that follow.

The neuro-fuzzy model comprises two main stages: learning and prediction. At the *learning stage*, the model trains itself using patient records for which the pathological stage is known, and at the *prediction stage* the model predicts the pathological stage using the knowledge which has been obtained during the learning stage. The following subsections describe the processes that are involved during the learning and prediction stages.

### System Inputs

At the *learning stage*, the neuro-fuzzy predictor learns (i.e. trains itself) using existing patient record data in order to create the knowledge which will be used (during the *prediction stage*) to make predictions on new, and previously unseen, data. During the learning stage, the system takes as input data relating to each patient’s clinical features (i.e. age at diagnosis, PSA, biopsy Primary Gleason pattern, biopsy Secondary Gleason pattern, and clinical T stage) and known pathological stage results (i.e. known outputs) that have been obtained during diagnosis. The system represents the inputs as a matrix *A* of size *n* × *m*, where *n* is the total number of patient records, and *m* is the total number of clinical features (i.e. system inputs, *m* = 5). The system represents the targets in the form of a *n* × 1 vector T, where each cell *t*_*i*_ holds the pathological T stage (pT) value for each patient record.

At the *prediction stage*, the system only requires as input an 1 × *m* vector holding the results of a patient’s clinical features (i.e. age at diagnosis, PSA, biopsy Primary Gleason pattern, biopsy Secondary Gleason pattern, and clinical T stage), and the system will return a value representing the likelihood of the patient having Extra-Prostatic Disease (i.e. pathological stage results).

### Data Normalisation

The age, PSA level, clinical T stage and pathological stage (pT) variables must be grouped before they are input into the fuzzy predictor. The normalisation of the values is described in the *Results* Section. The normalisation process is performed in order to ensure a balanced distribution among the data and to remove any outliers from the data which could affect the performance of the predictor algorithm.

### Fuzzy C-Means

Formally, let *A* = [*v*_1_, *v*_2_, *v*_3_, … , *v*_*n*_] be the [patient record cases]-by-[clinical features] matrix and let 2 ≤ *c* < *n* be an integer, where *c* is the number of clusters (i.e. classes) and *n* is the total number of patient record cases. In this particular prostate cancer application, *c* = 2 since we have two clusters: Organ-Confined Disease (OCD) and Extra-Prostatic Disease (ED). The Fuzzy C-Means (FCM) algorithm returns a list of cluster centers *X* = *x*_1_, … , *x*_*c*_ and a membership matrix *U* = *μ_i,k_* ∈ [0, 1]; *i* = 1, … , *n*; *k* = 1, … , *c*, where each element *μ*_*ik*_ holds the total membership of a data point *v*_*k*_ (i.e. patient record) belonging to cluster *c*_*i*_. FCM updates the centers of clusters Organ-Confined Disease and Extra-Prostatic Disease, and the membership grades for each data point, representing a patient record, by iteratively moving the cluster centers to the correct location within a data set. Essentially, this iteration process is based on minimizing an objective function which represents the distance from any given data point to a cluster center weighted by that data point’s membership grade. The objective function for FCM is a generalisation of Eq (1)

$$\begin{array}{c} J\left(U,c_{1},\ldots ,c_{c}\right)=\sum_{i=1}^{c}\sum_{k=1}^{N}\mu_{ik}^{m}\left|\right|v_{k}-x_{i}{\left|\right|}^{2},1\leq m\leq \infty \end{array}$$ (1)

where *μ*_*ik*_ represents the degree of membership of patient record *v*_*i*_ in the *ith* cluster; *x*_*i*_ is the cluster centre of fuzzy group *i*; || * || is the Euclidean distance between *ith* cluster and *jth* data point; and *m* ∈ [1, ∞] is a weighting exponent. The necessary conditions for function (1) to reach its minimum are shown in functions (2) and (3).

$$\begin{array}{c} c_{i}=\frac{\sum_{k=1}^{N}\mu_{ik}^{m}v_{k}}{\sum_{k=1}^{N}\mu_{i,k}^{m}}, \end{array}$$ (2)

$$\begin{array}{c} \mu_{ik}=\frac{1}{\sum_{k=1}^{c}}{\left(\frac{\left|\right|v_{k}-x_{i}\left|\right|}{\left|\right|v_{k}-x_{i}\left|\right|}\right)}^{2/(m-1)}, \end{array}$$ (3)

### Sugeno-Yusukawa Method

A collection of Takagi-Sugeno-Kang (TSK) rules [23], one for each cluster, for determining the membership of a patient record to a particular cluster are generated. This Sugeno-type Fuzzy Inference System (FIS) is generated using the FCM clustering algorithm. The number of clusters derived from the clustering process determines the number of rules and membership functions in the generated FIS. The FIS structure maps inputs through input membership functions and associated parameters, and then through output membership functions and associated parameters to outputs. The output FIS is passed into the Adaptive-Neuro Fuzzy Inference System (ANFIS) model [24] which then tunes the FIS parameters using the input/output training data in order to optimise the prediction model.

### Adaptive-Neuro Fuzzy Inference System

The Adaptive Neuro-Fuzzy Inference System (ANFIS) [24] combines Artificial Neural Networks and Fuzzy Logic algorithms. ANFIS creates a fuzzy inference system with membership functions that are generated by adaptive backpropagation learning. The architecture of a Type-3 ANFIS, which is the ANFIS used in the proposed model, is explained in [24]. The following is a brief description of ANFIS and is based on [25]. ANFIS consists of five layers. In layer 1, each node generates a membership grade of a linguistic variable (in the prostate cancer scenario, linguistic variables are the staging classes, i.e. Organ-Confined Disease and Extra-Prostatic Disease) using a membership function. The Gaussian membership function is used within the neuro-fuzzy model. Layer 2 calculates the firing strength of each rule, and layer 3 calculates the ratio of each rule’s firing strength to the total of all firing strengths. At layer 4, the contribution of each rule toward the overall output is computed, and, finally, layer 5 calculates the overall output as the summation of the contribution from each rule. During the learning process, ANFIS adapts the parameters associated with the membership functions and tunes them using a gradient vector which, given a set of parameters, measures the performance of the system on the basis of how well it models input and output data. ANFIS has been used in conjunction with FCM, and thus the FIS returned from FCM clustering is input into the ANFIS, and the FIS parameters are tuned using the input/output training data in order to optimise the prediction model.

The training process stops whenever the designated epoch number is reached, or the error goal is achieved. The performance of ANFIS is evaluated using the array of root mean square errors (difference between the FIS output and the training data output) at each epoch. Thus, the membership degree of a patient record into a particular cluster (e.g. Extra-Prostatic Disease), determines how close a prediction is to the next cluster (e.g. Organ-Confined Disease). In simple terms, let *c*_*a*_ and *c*_*b*_ be cluster Extra-Prostatic Disease and cluster Organ-Confined Disease respectively, a patient record *v*_*k*_ can belong to cluster *c*_*a*_ such that *v*_*k*_ ∈ *c*_*a*_, or it can belong in the intersection area between two clusters such that *v*_*k*_ ∈ *c*_*a*_ ∧ *v*_*k*_ ∈ *c*_*b*_.

### Neuro-Fuzzy Predictor

The neuro-fuzzy predictor takes as input a vector *X*_*i*_ of size 1 × *m*, where *m* is the total number of clinical features, hence 1 × 5 and the patient’s record is clustered as Organ-Confined Disease or Extra-Prostatic Disease, via the FCM clustering algorithm [26]. The predetermined Takagi-Sugeno-Kang (TSK) rules [23] are then applied in order to evaluate the degree of membership of the patient’s record to a particular cluster. The output is a numerical value representing the likelihood of a patient belonging to the Extra-Prostatic Disease cluster. This value is particularly useful when deciding on the suitable treatment to be offered to the patient. For example, treatment might be different if a patient is predicted as having Organ-Confined Disease with a value which leans more toward Extra-Prostatic Disease.

## Methods II—Other Computational Intelligence Approaches

### Artificial Neural Network Classifier

An Artificial Neural Network (ANN) can be trained to recognise patterns in data and this is a suitable approach for solving classification problems involving two or more classes. For the prostate cancer staging prediction problem, the ANN is trained to recognise the patients which have Organ-Confined Disease or Extra-Prostatic Disease. The pattern recognition neural network used was a two-layer feedforward network, in which the first layer has a connection from the network input and is connected to the output layer which produces the network’s output. A *log-sigmoid transfer function* was embedded in the hidden layer, and a *softmax transfer function* was embedded in the output layer.

A neuron has R number of inputs where R is the number of elements in an input vector. Let an input vector X be a patient record *X*_*i*_ belonging to a class Organ-Confined Disease or Extra-Prostatic Disease. Each input *X*_*i*_ is weighted with an appropriate weight *w*. The sum of the weighted inputs and the bias forms the input to the transfer function *f*. Neurons can use a differentiable transfer function *f* to generate their output. The Log-Sigmoid function which generates outputs between 0 and 1 as the neuron’s net input goes from negative to positive infinity was used. The Softmax neural transfer function was used to calculate a layer’s output from its net input. Softmax functions convert a raw value into a posterior probability and this provides a measure of certainty. The number of hidden neurons is set to 5 in order to match the number of inputs. The number of output neurons is set to 2, which is equal to the number of elements in the target vector (the number of classes, Organ-Confined Disease and Extra-Prostatic Disease). The maximum number of epochs (repetitions) was set to *ϵ* = 200 and in order to avoid over-fitting, training stops when the maximum number of epochs is reached. The ANN was trained using the Scaled Conjugate Gradient (SCG) for Fast Supervised Learning which is suitable for large-scale problems [27]. The process of training the ANN involves tuning the values of the weights and biases of the network in order to optimise network performance which is measured by the mean squared error network function.

### Naive Bayes Classifier

Although the Naive Bayes classifier is designed for use when predictors within each class are independent of one another within each class, it is known to work well even when that independence assumption is not valid. The Naive Bayes classifies data in two steps. The first step is the training (i.e. learning) step which uses the training data, which are patient cases and their corresponding pathological cancer stage (i.e. Organ-Confined Disease or Extra-Prostatic Disease), to estimate the parameters of a probability distribution, assuming predictors are conditionally independent given the class. The second step is the prediction step, during which the classifier predicts any unseen test data and computes the posterior probability of that sample belonging to each class. It subsequently classifies the test data according to the largest posterior probability. The following Naive Bayes description is based on that presented by Han et. al [8].

Let *P*(*c*_*i*_|*X*) be the posterior probability that a patient record *X*_*i*_ will belong to a class *c*_*i*_ (class can be Organ-Confined Disease or Extra-Prostatic Disease), given the attributes of vector *X*_*i*_. Let *P*(*c*_*i*_) be the prior probability that a patient’s record will fall in a given class regardless of the record’s characteristics; and *P*(*X*) is the prior probability of record *X*, and hence the probability of the attribute values of each record. The Naive Bayes classifier predicts that a record *X*_*i*_ belongs to the class *c*_*i*_ having the *highest posterior probability*, conditioned on *X*_*i*_ if and only if *P*(*c*_*i*_|*X*) > *P*(*c*_*j*_|*X*) for 1 ≤ *j* ≤ *m*, *j* ≠ *i*, maximising *P*(*c*_*i*_|*X*). The class *c*_*i*_ for which *P*(*c*_*i*_|*X*) is maximised is called the *maximum posteriori hypothesis* and estimated using formula (4)

$$\begin{array}{c} P\left(c_{i}|X\right)=\frac{P\left(X|c_{i}\right)P\left(c_{i}\right)}{P(X)}. \end{array}$$ (4)

To predict the class label of a given record *X*_*i*_, *P*(*X*|*c*_*i*_)*P*(*c*_*i*_) is evaluated for each class *c*_*i*_. The classifier predicts that the class label of record *X*_*i*_ is the class *c*_*i*_ if and only if

$$\begin{array}{c} P\left(X|c_{i}\right)P\left(c_{i}\right)>P\left(X|c_{j}\right)P\left(c_{j}\right) \end{array}$$ (5)

for 1 ≤ *j* ≤ *m*, *j* ≠ *i*.

The Naive Bayes outcome is that each patient’s record, which is represented as a vector *X*_*i*_, is mapped to exactly one class *c*_*i*_, where *c*_*i*_ = 1, … , *n* where *n* is the total number of classes, i.e. *n* = 2. The Naive Bayes classification function can be tuned on the basis of an assumption regarding the distribution of the data. Experiments were conducted using two methods of density estimation: the first one assumes normality and models each conditional distribution with a single Gaussian; and the second uses nonparametric kernel density estimation. Hence, the Naive Bayes classifier was tuned using two functions: a Gaussian distribution (GD) and the Kernel Density Estimation (KDE). The Gaussian distribution assumes that the variables are conditionally independent given the class label and thereby exhibit a multivariate normal distribution, whereas *kernel density estimation* does not assume a normal distribution and hence it is a non-parametric technique.

### Support Vector Machine Classifier

The Support Vector Machine (SVM) classification method uses nonlinear mapping to transform the original training data (i.e. the patient dataset) into a higher dimensional feature space. It then determines the best separating hyperplane, which serves as a boundary separating the data from two classes. The best separating hyperplane for a Support Vector Machine means the one with the largest margin between the two classes. The bigger the margin, the better the generalisation error of the linear classifier is defined by the separating hyperplane. Support vectors are the points that reside on the canonical hyperplanes and are the elements of the training set that would change the position of the dividing hyper plane if removed. As with all supervised learning models, a support vector machine is initially trained on existing data records, after which the trained machine is used to classify (predict) new data. Various Support Vector Machine kernel functions can be utilised to obtain satisfactory predictive accuracy.

The Support Vector Machine finds the Maximum Marginal Hyperplane (MMH) and the support vectors using a Lagrangian formulation and solving the equation using the Karush-Kuhn-Tucker (TTK) conditions, details of which can be found in [28]. Once the Support Vector Machine has been trained, the classification of new unseen patient records is based on the Lagrangian formulation. For many ‘real-world’ practical problems, using the linear boundary to separate the classes may not reach an optimal separation of hyperplanes. However, Support Vector Machine kernel functions which are capable of performing linear and nonlinear hyperplane separation exist. The outcome of applying the Support Vector Machine for prediction is that each patient record, represented as a vector *X*_*i*_, is mapped to exactly one class label *y*_*i*_, where *y*_*i*_ = ±1, such that (*X*_1_,*y*_1_), (*X*_2_, *y*_2_), …(*X*_*m*_, *y*_*m*_), and hence *y*_*i*_ can take one of two values, either −1 or +1 corresponding to the classes Organ-Confined Disease and Extra-Prostatic Disease. Further details on the Support Vector Machine can be found in [29], and [30].

## Results I: Dataset Analysis

### Dataset Description

The Cancer Genome Atlas (TCGA) Research Network provides datasets for cancer patients which are made open to the public through the Data Coordinating Center and the TCGA Data Portal. The prostate cancer dataset obtained from the TCGA contains records collected from 399 patients diagnosed with a type of *Prostate Adenocarcinoma Acinar*, during the years 2000–2013. All patients had prostate needle core biopsies for diagnosis before they underwent prostatectomy, and all patients had undergone a prostatectomy. The variables selected from the dataset were those that are used for performing prostate cancer stage predictions by clinicians, and which are also required for undertaking staging prediction using the AJCC pTNM Nomogram [21]—namely, biopsy Primary and Secondary Gleason patterns, pre-treatment PSA level, patient’s age at diagnosis, clinical T stage, and pathological T stage.

The age and PSA variables were categorically divided into groups that were chosen in order to ensure a balanced distribution between the data, as described later in this section. Table 4 provides statistics about the variables before they were categorised.

**Table 4:** Dataset Statistics.

Statistics of variables before categorisation				
	Minimum	Maximum	Mean	Standard deviation
**Primary Gleason pattern**	3	5	3.54	0.60
**Secondary Gleason pattern**	3	5	3.74	0.69
**PSA level (ng/mL)**	0.70	107.00	9.84	11.25
**Age at Diagnosis**	41.10	78.00	59.88	6.92
**Clinical T**	1.00	5.00	2.19	1.45
**Pathological T stage**	1.00	2.00	1.55	0.50

The Primary and Secondary Gleason pattern variables (see Table 5) did not require any modification, as they are already categorically divided into three groups.

**Table 5:** Primary and Secondary Gleason pattern groups.

**Primary Gleason pattern groups**	**Frequency count**	**Proportion of patients(%)**
**3**	205	51.4
**4**	173	43.4
**5**	21	5.3
**Total**	399	100.0
**Secondary Gleason pattern groups**	**Frequency count**	**Proportion of patients(%)**
**3**	159	39.8
**4**	185	46.4
**5**	55	13.8
**Total**	399	100.0

**Fig 2:**
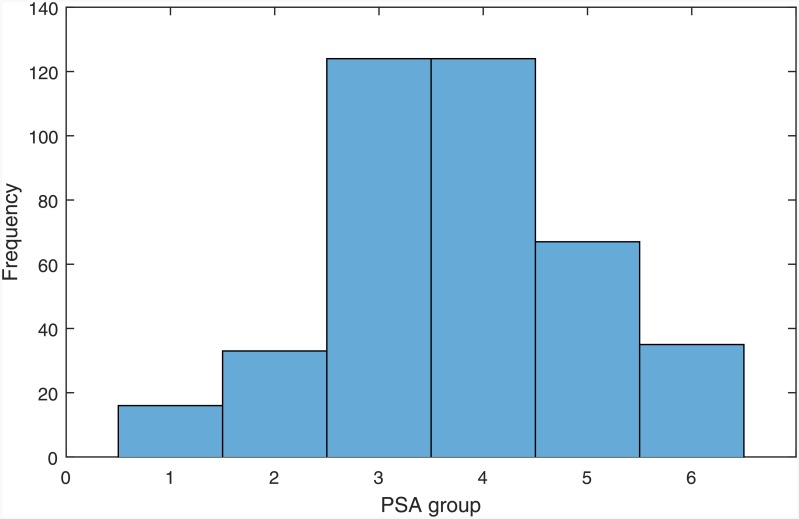
Histogram of grouped PSA values.

The variables of pre-treatment total serum PSA levels and age were categorically divided into groups, as described in Tables 6 and 7. Table 6 shows how the PSA values have been grouped, the number of cases in each group (i.e. count), and their percentage. The histogram in Fig 2 illustrates the frequency distributions of the grouped PSA values.

**Table 6:** PSA groups.

PSA group	PSA range	Frequency count	Proportion of patients (%)
**1**	0–2.5 ng/mL	16	4.01
**2**	2.6–4.0 ng/mL	33	8.27
**3**	4.1–6.0 ng/mL	124	31.08
**4**	6.1–9.9 ng/mL	124	31.08
**5**	10–19 ng/mL	67	16.79
**6**	≥ 20 ng/mL	35	8.77

**Table 7:** Age groups.

Age group	Age range	Frequency count	Proportion of patients (%)
**1**	< 25	0	0
**2**	25–29	0	0
**3**	30–34	0	0
**4**	35–39	0	0
**5**	40–44	5	1.25
**6**	45–49	22	5.51
**7**	50–54	68	17.04
**8**	55–59	97	24.31
**9**	60–64	100	25.06
**10**	65–69	76	19.05
**11**	> 70	31	7.77

Table 7 displays how the age values have been grouped, the number of patient cases in each group (i.e. count), and the percentage of cases. Although it is very unlikely for a patient to have prostate cancer under the age of 35, there is still a possibility, and for this reason, groupings 1 to 4 have been formed. However, none of the patient cases fall in this category in the particular dataset which was used in the current study. This does not affect the performance of the system in any way, and these groupings were included in order to create a comprehensive prediction system.

The clinical T and pathological T stage variable values were grouped as shown in Tables 8 and 9 respectively. The clinical T stage variable values were grouped in such a way so as to match the groups that are presented on the TNM nomogram. Group 1 includes T stages T1a-c, which reflects the fact that tumor is present in one or both lobes by needle biopsy, but is not identifiable on the basis of palpation or is reliably visible by imaging. Group 2 includes clinical T stages in which the cancer is unilateral, meaning that it is located on one-half of one side or less; group 3 is unilateral and involves more than one-half of one side, but not both sides; group 4 is when the cancer is in the form of bilateral disease which is located on both sides of the prostate; and group 5 is an Extra-Prostatic Disease, meaning that the cancer has started to spread (or has spread) to the bladder neck, rectum, and/or nearby organs.

**Table 8:** Clinical T stage groups.

Clinical T group	Clinical T stage	Frequency count	Proportion of patients (%)
**1**	T1(a-c)	204	51.13
**2**	T2a	53	13.28
**3**	T2b	53	13.28
**4**	T2c	42	10.53
**5**	T3a	29	7.27
**5**	T3b	16	4.01
**5**	T4	2	0.50
	**Total**	399	100.00

**Table 9:** Pathological T (pT) stage groups.

pT group	Pathological T (pT) stage	Frequency count	Proportion of patients (%)	OCD or ED
**1**	T2(unknown if a or b)	1	0.25	OCD
**1**	T2a	14	3.51	OCD
**1**	T2b	47	11.78	OCD
**1**	T2c	117	29.32	OCD
**2**	T3a	142	35.59	ED
**2**	T3b	72	18.05	ED
**2**	T4	6	1.50	ED
	**Total**	399	100.00	

The independent variable (i.e. predictor variable) of the dataset is the pathological T (pT) stage, whose values have been categorised as 1 for Organ-Confined Disease (OCD) and 2 for Extra-Prostatic Disease (ED). There is no pathologic pT1 classification, and pathological T stage values in the pT2 range indicate an Organ-Confined Disease and pathological T stage values in the range of 3–4 indicate an Extra-Prostatic Disease.

Given that the aim of the model was to predict whether a patient has organ-confined disease (OCD, TNM pathological stage *pT*2) or extra-prostatic disease (ED, TNM pathological stage > *pT*2), and not the likelihood of cancer-related death (DOD, dead of disease), the pT3 and pT4 groupings were consolidated. T3a, T3b and T4 disease are all considered as being ‘high-risk disease’ and strongly considered for active treatment, whereas those with lower categories of disease might instead be considered for active surveillance. Table 9 includes the groupings of the pathological T stage values.

Tables 10 and 11 present a sample of the data before and after data normalisation (i.e. categorically divided into groups), respectively. As previously mentioned, the values of Primary and Secondary Gleason patterns did not require any transformation, as they were already grouped.

**Table 10:** Before data normalisation.

Case No.	Primary Gleason Pattern	Secondary Gleason Pattern	PSA	Age	Clinical T stage	Pathological (pT) stage
**1**	3	3	1.00	51.6	T2b	T2a
**2**	3	3	1.70	77.0	T2b	T2c
**3**	3	3	2.05	55.2	T2a	pT2b
**4**	3	3	2.09	61.1	T1c	pT2b
**5**	3	3	2.20	57.0	T1c	T3a
**n**	…	…	…	…	…	…

**Table 11:** After data normalisation.

Case No.	Primary Gleason Pattern	Secondary Gleason Pattern	PSA group	Age group	Clinical T group	Pathological (pT) group
**1**	3	3	1	7	3	1
**2**	3	3	1	11	3	1
**3**	3	3	1	8	2	1
**4**	3	3	1	9	1	1
**5**	3	3	1	8	1	2
**n**	…	…	…	…	…	…

### Age at Diagnosis and its Association with PSA Values

**Fig 3:**
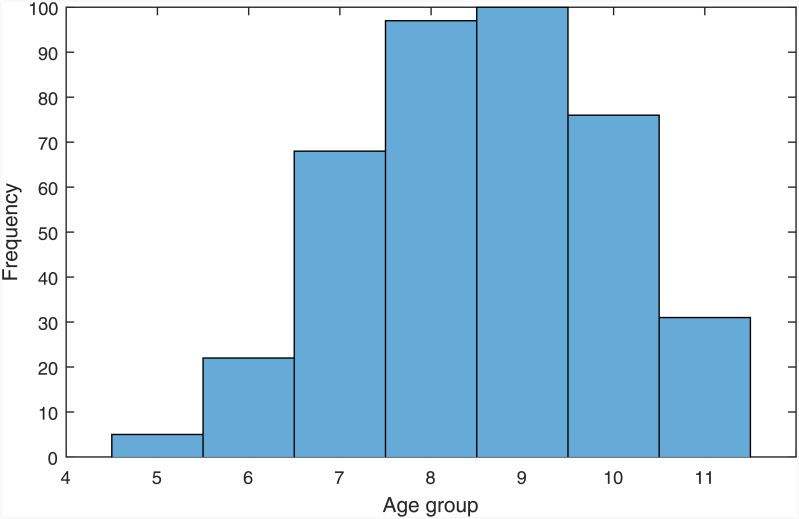
Histogram of grouped age values.

Evidence indicates that age could be a contributing factor to increased PSA levels [16], and for this reason it is informative to investigate whether there are any associations between PSA levels and age in the dataset. The histograms illustrating the frequency distributions of the grouped PSA levels and age are illustrated in Figs 2 and 3 respectively. The mean *age at diagnosis* of patients was 59.88 ± 6.92, and the mean *pre-treatment PSA level* was 9.84 ± 11.25. Table 12 shows the mean and standard deviation PSA values of each age group.

**Table 12:** PSA levels categorised by age group.

Age group	Patient count	PSA mean	Standard deviation of PSA values
**5**	5	4.40	1.14
**6**	22	4.14	0.89
**7**	68	3.54	1.23
**8**	97	3.75	1.20
**9**	100	3.74	1.14
**10**	76	3.75	1.21
**11**	31	3.81	1.56
**Total**	399	3.75	1.21

To investigate if there is a statistically significant association between age and PSA levels, a Spearman’s rho correlation test was performed. The results revealed no significant associations among age and PSA levels (*r* = 0.003, *p* = 0.948), at least over the age ranges (40 to 78) that were included in the dataset which was used. A one-way ANOVA was also conducted to test for statistically significant differences between the PSA values of the various age groups. The test indicated that there were no statistically significant differences among any of the groups, *F*(6,392) = 0.956, *p* = 0.455, *p* > 0.05). Since age and PSA values are not associated, then both variables were used as inputs by the prediction system.

### Analysis of the Clinical T Stage Values

The clinical T stage values are the results of a Digital Rectal Examination (DRE) test. Table 8 shows the total number of patients for each clinical T stage. As shown in Table 8, the clinical stage values of T1 (*n* = 204, 51.13%), T2a (*n* = 53, 13.28%), T2b (*n* = 53, 13.28%), T2c (*n* = 42, 10.53%), denote an Organ-Confined Disease cancer stage, and the T3a (*n* = 29, 7.27%), T3b (*n* = 16, 4.01%), T4 (*n* = 2, 0.50%) denote an Extra-Prostatic Disease cancer stage. At a clinical stage, a total of 352 patients (88.22%) exhibited Organ-Confined Disease, and a total of 47 patients exhibited Extra-Prostatic Disease (11.78%). Clearly, the results of the clinical T test alone is not as reliable as the pathological T (pT) test for determining the stage of prostate cancer. This is evident since, at the pathological stage (i.e. diagnosis stage), out of the 399 patients, a total of 44.86% (*n* = 179) had Organ-Confined Disease, and 55.14% (*n* = 220) had Extra-Prostatic Disease, meaning that 43.36% (n = 173) patients with Extra-Prostatic Disease were misdiagnosed as having Organ-Confined Disease. DRE is not always a reliable test, since the location of the tumor within the prostate might influence the capacity to feel it.

### Analysis of the Pathological T (pT) Stage Values

Table 9 shows the total number of patients for each pathological T stage. As shown in Table 9, the pathological T stage values of T2a-c denote an Organ-Confined Disease cancer stage, and the pathological T stage values of T3a,b,T4 denote an Extra-Prostatic Disease cancer stage. Of the 399 patients, a total of 44.86% (*n* = 179) exhibited Organ-Confined Disease, and 55.14% (n = 220) exhibited Extra-Prostatic Disease.

Table 13 shows the relationship between the prediction variables and prostate cancer with Organ-Confined Disease and Extra-Prostatic Disease. A one-way ANOVA test revealed significant differences between the means of the two groups for the biopsy Primary Gleason pattern (*F*(1,397) = 7.87, *p* = 0.005, *p* < 0.05), biopsy Secondary Gleason pattern (*F*(1,397) = 5.83, *p* = 0.016, *p* < 0.05), and clinical T stage (*F*(1,397) = 5.062, *p* = 0.025, *p* < 0.05) variables. These results were expected, since Gleason 1 grading of the biopsy determines the aggressiveness of the cancer, and hence patients with Extra-Prostatic Disease are more likely to have a higher value in this category than patients with Organ-Confined Disease. The same applies for PSA levels, as these tend to increase with progressive and more extensive disease. In addition, the clinical T stage values determine the outcome of a physical examination and the higher the number of the clinical T stage value, the more the disease has progressed. Interestingly, in this particular dataset, no statistically significant differences among the means values of the pre-treatment PSA levels (*F*(1,397) = 0.037, *p* = 0.848, *p* > 0.05) and age (*F*(1,397) = 0.614, *p* = 0.434, *p* > 0.05) variables were apparent. Although the mean age of patients with Extra-Prostatic Disease was higher than that of patients with Organ-Confined Disease, this difference is not statistically significant. Also, there were no statistically significant differences among the mean PSA values of patients with Extra-Prostatic Disease and Organ-Confined Disease. In summary, the mean values of all but the PSA and age variables were significantly higher in the Extra-Prostatic Disease than in the Organ-Confined Disease groups.

**Table 13:** Mean and Standard deviation values for Organ-Confined Disease (OCD) and Extra-Prostatic Disease (ED) groups diagnosed at the Pathological stage.

	Groups		
Variables	OCD	ED	*p*
	n = 179	n = 220	
**Primary Gleason pattern**	3.45 ± 0.52	3.61 ± 0.64	0.005
**Secondary Gleason pattern**	3.65 ± 0.64	3.81 ± 0.71	0.016
**Pre-treatment PSA level (ng/mL)**	3.76 ± 1.26	3.74 ± 1.17	0.848
**Age at diagnosis (groups)**	8.49 ± 1.37	8.60 ± 1.40	0.434
**Clinical T stage**	2.01 ± 1.36	2.33 ± 1.51	0.025

## Results II: Pathological Stage Prediction Using the Neuro-Fuzzy Model

### Experiment Methodology

Having analysed the dataset and, as appropriate, grouped the data, the next step is to explain how the transformed data will be input into the neuro-fuzzy model and the other models which will be used for the comparison process. In particular, the performance of the neuro-fuzzy model is compared to other computational intelligence based approaches, namely the Artificial Neural Network, Fuzzy C-Means, Support Vector Machine, and the Naive Bayes classifier. All of these classifiers are suitable for solving prediction problems, as is the American Joint Committee on Cancer (AJCC) pTNM Nomogram [21] which is a statistical approach that is commonly adopted by clinicians for predicting prostate cancer staging. For predicting the pathological stage of cancer, the AJCC pTNM Nomogram uses all variables except the age at diagnosis variable. These variables are found in Table 13.

### System Inputs

**Fig 4:**
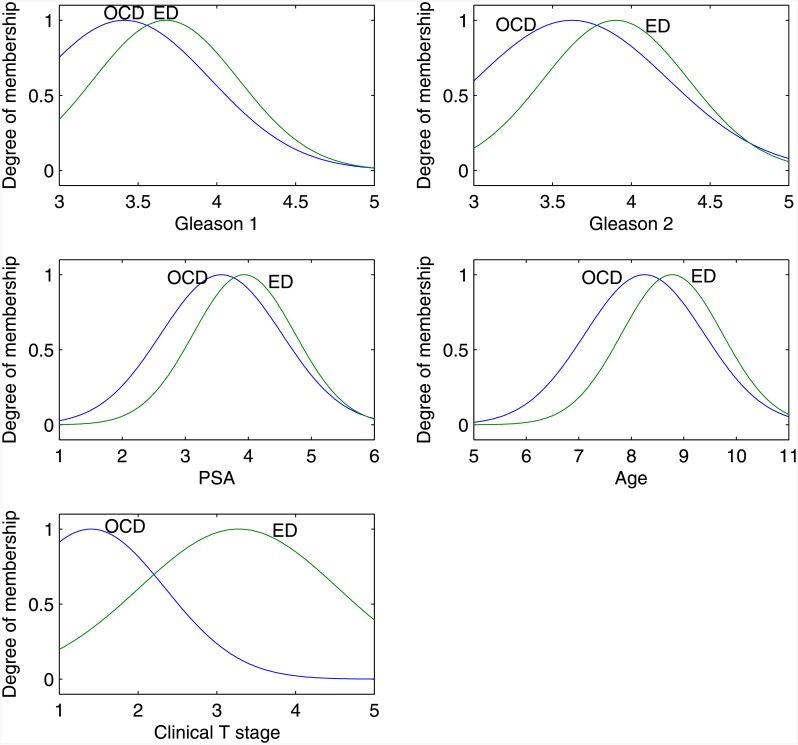
Neuro-Fuzzy System Membership Functions: Gleason 1 is Primary Gleason Pattern; Gleason 2 is Secondary Gleason pattern; PSA is Prostate Specific Antigen; Age represents the Age group; and clinical T stage is the result of the Digital Rectal Examination. OCD is Organ-Confined Disease and ED is Extra-Prostatic Disease.

All classification models take as input a matrix A of size *n* × *m*, where *n* is the total number of patient records and *m* is the total number of clinical features, hence 399 × 5; and a *n* × 1 vector T, where *n* = 399 and each cell *t*_*i*_ holds the pathological T (pT) stage value for each patient record. Table 11 shows the first five records of matrix A after normalising the input values, in which the first 5 columns are the inputs and the last column pathological T (pT) stage holds the target output values. The dataset (*n* = 399) was separated into two subsets, a training subset and a validation subset, and the same subsets were used across the models undergoing evaluation in order to ensure a fair comparative evaluation. The training subset comprised 266 (66.6%) records, which were used for training each model. The validation subset comprised 133 (33.3%) records and these were used for determining the predictive accuracy of each model (i.e. validating its performance). Of the 133 records used for validation, 66 (49.62%) records corresponded to patients with Organ-Confined Disease, and 67 (50.38%) records corresponded to patients with Extra-Prostatic Disease. Fig 4 shows a set of Gaussian membership functions generated by the proposed system, for each input data. The input comprised of 5 inputs given by two external markers (Organ-Confined Disease, and Extra-Prostatic Disease). Observing the membership curves found in Fig 4, reveals a consistency among them—the results of tests corresponding to patients with Extra-Prostatic Disease fall in a higher range than the results of patients with Organ-Confined Disease.

The performance of the proposed neuro-fuzzy model was compared to that of an Artificial Neural Network, Fuzzy C-Means, Support Vector Machine, and the Naive Bayes classifiers; and the AJCC pTNM Nomogram statistical approach [21] which can also be considered as a classifier. The subsections below give a brief introduction to each classification model and details on how the parameters of each model were appropriately tuned in order to report their best performance.

### Performance Evaluation Measures

The evaluation measures that were adopted for assessing the performance of each approach for predicting the pathological stage of patients are *Sensitivity* and *Specificity*. These statistical measures are used for evaluating the performance of binary classification tests and are suitable since the aim is to measure the performance of each system in distinguishing Extra-Prostatic Disease from the Organ-Confined Disease.

Sensitivity (i.e. True Positive Rate) measures the proportion of actual positives which are correctly identified as such (e.g. the percentage of Extra-Prostatic Disease patients who are correctly identified as Extra-Prostatic Disease). Specificity (i.e. True Negative Rate) measures the proportion of negatives which are correctly identified as such (e.g. the percentage of patients with Organ-Confined Disease who are correctly identified as not having Extra-Prostatic Disease). A perfect system would return 100% sensitivity (e.g., all patients with Extra-Prostatic Disease are classed as Extra-Prostatic Disease) and 100% specificity (e.g. all patients with Organ-Confined Disease are not classed as Extra-Prostatic Disease). The following notation relates to the evaluation measures.

- Let |*TP*| be the total number of patients with Extra-Prostatic Disease correctly classified as Extra-Prostatic Disease.
- Let |*TN*| be total the number of patients with Organ-Confined Disease correctly classified as Organ-Confined Disease.
- Let |*FP*| be the total number of patients with Organ-Confined Disease incorrectly classified as Extra-Prostatic Disease.
- Let |*FN*| be the total number of patients with Extra-Prostatic Disease incorrectly classified as Organ-Confined Disease.
- Let |*P*| be the total number of Extra-Prostatic Disease cases that exist in the dataset, where |*P*| = |*TP*| + |*FN*|.
- Let |*N*| be the total number of patients with Organ-Confined Disease that exist in the dataset, where |*N*| = |*FP*| + |*TN*|.

The functions for the Sensitivity and Specificity evaluation measures are presented in Functions (6) and (7) respectively.

$$\begin{array}{c} Sensitivity\left(k\right)=\frac{\left|TP\right|}{\left|TP|+|FN\right|},\in \left[0,1\right]. \end{array}$$ (6)

$$\begin{array}{c} Specificity\left(k\right)=\frac{\left|TN\right|}{\left|TN|+|FP\right|},\in \left[0,1\right]. \end{array}$$ (7)

The closer the values of Sensitivity and Specificity are to 1.0, the better the detection performance of the system.

Evaluation measures based on the Receiver Operating Characteristic (ROC) curve analysis are fundamental in clinical research. ROC curves are used to determine the performance of the systems, and they can be used to establish a cutoff value for optimal performance of each system. The ROC curve is a graph of sensitivity (y-axis) against 1-specificity (x-axis) across different cut-off points. The area under the ROC curve (AUC) is a reflection of how good the system’s performance is at distinguishing (or discriminating) between patients with and without Extra-Prostatic Disease—the larger the area, the better the performance. The aim is to determine the cutoff point for which the classifier returns the high number of true positives and the low number of false positives. Maximizing sensitivity corresponds to some large True Positive Rate (y-axis value) on the ROC curve, and maximizing specificity corresponds to a small False Positive Rate value (x-axis value) on the ROC curve. Thus, the optimal cutoff value is to the upper left corner of the chart, the higher the overall accuracy of the classifier. Hence, a system which perfectly discriminates Organ-Confined Disease and Extra-Prostatic Disease has 1.0 (or 100%) sensitivity and 1.0 (or 100%) specificity.

### Comparison of the Neuro-Fuzzy Model with Other Methods

The aim of the evaluation is to measure the ability of each system to predict the pathological stage of patients. The results presented in this section are those for validating the system, as these determine the true ability of a system to discriminate Organ-Confined Disease from Extra-Prostatic Disease using the knowledge which has been acquired by the system during the training (i.e. learning) process. To perform these evaluations, the actual outputs returned by each system during the validation stage were compared against the targets (i.e. known) outputs.

**Fig 5:**
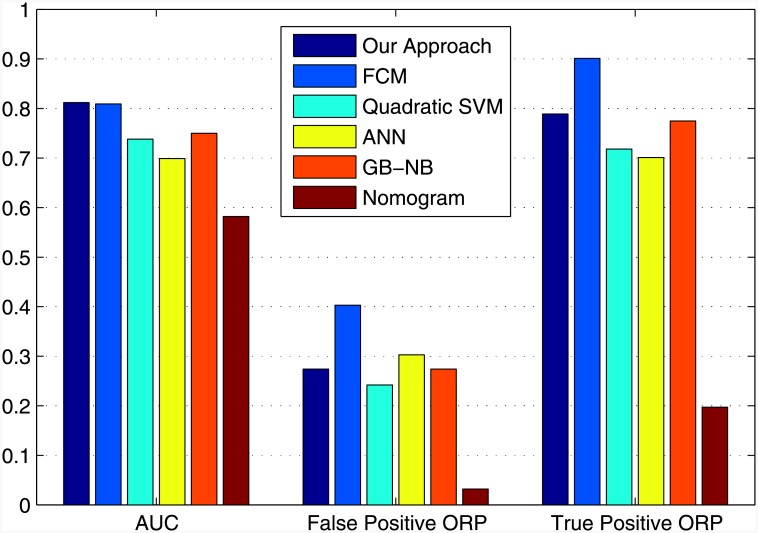
Performance Comparison.

**Fig 6:**
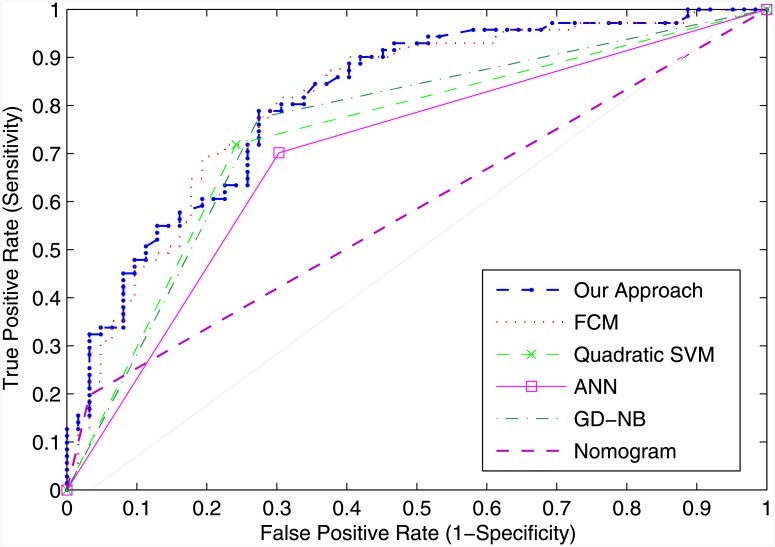
ROC Curves: Performance Comparison.

The results of the comparisons are shown in Table 14 and illustrated in Fig 5. The ROC curves for all systems are shown in Fig 6. The cutoff points of each classifier are presented in rows, Optimal ROC point False Positive Rate and Optimal ROC point True Positive Rate, of Table 14 and were computed with the alpha values set to *α* = 0.05 (95% Confidence Interval). The best system would return the largest AUC, a high number of true positives, and a low number of false positives.

**Table 14:** Performance evaluation.

	Performances based on ROC evaluation measurements					
	Neuro-Fuzzy (Our approach)	FCM	Quadratic-SVM	ANN	GB-NB	AJCC pTNM Nomogram
**Area Under the Curve (AUC)**	0.812	0.809	0.738	0.699	0.750	0.582
**Optimal ROC point FPR**	0.274	0.403	0.242	0.303	0.274	0.032
**Optimal ROC point TPR**	0.789	0.901	0.718	0.701	0.775	0.197
**Asymp. Sig. (McNemars)**	1.000	0.868	0.499	1.000	1.000	0.000

The Support Vector Machine was trained using the Linear kernel function, Quadratic, Gaussian Radial Basis (GRB), Multilayer Perceptron kernel (MP) functions. The results of testing the performance (i.e. validation) of the Support Vector Machine using the various kernel functions are presented in Table 15. The results show that the Quadratic-Support Vector Machine has the largest AUC (*AUC* = 0.738), thereby outperforming all other functions.

**Table 15:** Support Vector Machine(SVM) performance evaluation when applying various kernel functions.

	Kernel Function			
Evaluation Measure	Linear	Quadratic	GRB	MP
**Specificity (TNR)**	0.758	0.758	0.661	0.597
**Sensitivity (TPR)**	0.704	0.718	0.747	0.690
**Area Under the Curve**	0.731	0.738	0.704	0.644
**Optimal ROC point FPR**	0.242	0.242	0.339	0.403
**Optimal ROC point TPR**	0.704	0.718	0.747	0.690

The Naive Bayes classifier results, when tuned using the Gaussian Distribution (i.e. normal distribution) and Kernel Density Estimation functions, are presented in Table 16. The results revealed that the Gaussian Naive Bayes (GD-NB) classifier returned a larger AUC (*AUC* = 0.750), thereby outperforming the Kernel Density Estimation Naive Bayes (KDE-NB) classifier (*AUC* = 0.696).

**Table 16:** Naive Bayes(NB) performance evaluation using the Gaussian distribution and Kernel Density Estimation functions.

	Type of function	
Evaluation Measure	GD-NB	KDE-NB
**Specificity (TNR)**	0.726	0.645
**Sensitivity (TPR)**	0.745	0.747
**Area Under the Curve**	0.750	0.696
**Optimal ROC point FPR**	0.274	0.355
**Optimal ROC point TPR**	0.775	0.747

The best Support Vector Machine and Naive Bayes classifiers are included in Table 14 which contains all of the results. The results of the comparison revealed that the proposed neuro-fuzzy system, at its optimal point, returns the largest AUC, with a low number of false positives (*FPR* = 0.274, *TPR* = 0.789, *AUC* = 0.812). Although the FCM classifier returned the highest number of true positives, it returned a very high number of false positives (*FPR* = 0.403, *TPR* = 0.901, *AUC* = 0.809). For these reasons, FCM cannot be considered as the optimum classifier. The Quadratic-Support Vector Machine, ANN, and GB-NB did not perform as well as the neuro-fuzzy approach—they returned a smaller AUC, and a lower optimal TPR. The AJCC pTNM Nomogram performed the worst, with the smallest AUC (0.582), and the lowest number of TPR (0.197) at the optimal ROC point. The proposed neuro-fuzzy system therefore outperformed all other systems.

Finally, Table 14 shows the results of the McNemar test which was applied to investigate whether any statistically significant differences exist among each system’s target outputs and the actual outputs. The McNemar test is a statistical test which is applied on paired nominal data. It uses an approximate chi-square test of goodness to test the null hypothesis, i.e. there are no significant differences among targets and outputs. Each pair comprised of the actual and predicted values of each system. A good system will not return a statistically significant difference (i.e. *p* > 0.05) amongst its predicted outputs and the actual target outputs (known outputs). The results revealed that there were no statistically significant differences among the actual and predicted outputs of the proposed neuro-fuzzy approach (*p* = 1.000, *p* > 0.05); the FCM classifier, (*p* = 0.868, *p* > 0.05); the Quadratic-SVM, (*p* = 0.499, *p* > 0.05); the ANN (*p* = 1.000, *p* > 0.05); and the GB-NB, (*p* = 1.000, *p* > 0.05). However, there was a statistically significant difference among the outputs of the AJCC pTNM Nomogram against targets (*p* = 0.00, *p* < 0.05).

## Discussion and Conclusion

At the clinical prostate cancer staging process, the patient undergoes various clinical tests for the prognosis of prostate cancer, and based on these tests, the clinician estimates (or predicts) how much the cancer has spread. It is only after surgery, and hence at the pathological stage, that it is possible to more accurately diagnose cancer and determine the extent of its spread beyond the prostate gland. The ability to predict that pathological stage of prostate cancer is important, as it allows clinicians to determine the best approach for treating and managing the disease.

Herein, we propose the application of a neuro-fuzzy based approach for the prediction of the pathological stage of prostate cancer. The algorithm is suitable for the particular problem due to the imprecision, and the uncertainty which is typically found in the results of the clinical tests which can be used for predicting the pathological stage of prostate cancer. The system input comprised of variables Primary and Secondary Gleason patterns, PSA levels, age at diagnosis, and clinical T stage. The output is the pathological stage of the cancer which can be either Organ-Confined Disease or Extra-Prostatic Disease. Experiments were performed using an existing and validated prostate cancer patient dataset comprising *n* = 399 patient records obtained from The Cancer Genome Atlas (TCGA) Research Network. The performance of the proposed neuro-fuzzy system was compared to other classifiers: the Artificial Neural Network, Fuzzy C-Means, Support Vector Machines, the Naive Bayes, and the AJCC pTNM Nomogram [21]. The results revealed that the proposed neuro-fuzzy system outperformed all other classifiers. Our results appear to be consistent to those of Castanho et al. [12] who have also developed genetic-fuzzy expert system for predicting whether prostate cancer if confined or not-confined. Their results have also revealed that computational intelligence approaches based on fuzzy algorithms are suitable for prostate cancer staging prediction, and exceed the performance of nomograms.

The algorithm proposed by Castanho et al. [12] tunes the membership functions using a genetic algorithm, whereas we have used the Adaptive Neuro Fuzzy Inference System (ANFIS) to optimise the membership functions. Furthermore, Castanho et al.’s [12] and our proposed system both aim to predict whether a patient has organ-confined disease (OCD, pathological stage pT2) or extra-prostatic disease (ED, pathological stage > *pT*2). Although both systems use pre-operative serum PSA, clinical stage, and primary and secondary Gleason grades of a biopsy to predict the pathological stage of prostate cancer, our system considers age as an additional input variable. Castanho et al.’s [12] genetic-fuzzy system achieved an Area Under the Curve of 0.824 which they compared against Partin probability tables which have been proposed by Makarov et al. [31], and which only achieved an Area Under the Curve of 0.693. Our proposed neuro-fuzzy approach achieved an Area under the curve of 0.812, and the AJCC nomogram achieved an Area Under the Curve of 0.582. These results approximate to those reported by Castanho et al. [12], and reveal a high degree of consistency among the two outcomes of the two studies, despite the fact that different datasets were used for each study. The nomograms used by Castanho et al., and the AJCC nomogram both use the TNM Classification of Malignant Tumors grading system [32]. A major limitation of the AJCC nomogram is that the biopsy Gleason 7 values are not split into 3 + 4 = 7 vs. 4 + 3 = 7 which have drastically different clinical outcomes. The proposed neuro-fuzzy model considers the Gleason Grades 3 + 4 and 4 + 3, and this was one of the reasons that it performed better than the AJCC nomogram.

A recent study by Tsao et al. [15] has also reported similar AUC values, to those returned by our model and that of Castanho et al. [12], when using the Partin probability tables proposed which have been proposed by Makarov et al. [31] to predict the pathological stage of prostate cancer in patients prior to receiving radical prostatectomy. Tsao et al. [15] developed an artificial neural network (ANN) model to predict the pathological stage of prostate cancer and evaluated the model on 299 patients, of whom 109 (36.45%) displayed prostate cancer with extra-capsular extension (ECE), and 190 (63.55%) displayed organ-confined disease (OCD). Overall, their results revealed that the ANN model (*AUC* = 0.795) significantly outperformed a Linear Regression statistical model (*AUC* = 0.746), and the Partin Tables (*AUC* = 0.695).

It should be noted that other predictors/nomograms consider features other than clinical stage, PSA, age, and biopsy Gleason grade. Some use the amount of tumor present, while others are starting to incorporate results of molecular analysis data, such as data from Prolaris [33] or oncotype measurements. Such models were not considered in the current study, as the relevant information is not available via the TCGA datasets and urologists predominantly use the Kattan preoperative nomogram [34] and the Partin Tables [35] for determining the likelihood of prostate cancer recurrence following radical prostatectomy, at least in the UK and Europe.

A study by Tamblyn et al. [36] which compared the Cancer of the Prostate Risk Assessment (CAPRA) score [37] against the Kattan (version 1998) [34] and Stephenson nomograms (version 2006) [38] revealed that the Kattan (version 1998) [34] tool was the best predictor of absolute risk of recurrence. Furthermore, a recent study by Boehm et al. [39] which compared three preoperative models, D’Amico [40], CAPRA [37] and Stephenson [38], revealed that these tools are reliable in North American patients, but have shortcomings for identifying patients at high risk of prostate cancer death in Europe. D’Amico [40] and CAPRA [37] include the amount of tumor detected on biopsy as part of their risk prediction algorithm and consider volume to have an influence on the risk of disease recurrence. However, from the evidence presented in Boehm et al. [39], this is not necessarily the case for non-US patients. Therefore, the precise influence of tumor volume on the risk of disease is inconclusive. Finally, although the volume of tumour per each core has been used to determine the significance of the tumor, Gleason 6 disease is still regarded as being a non-significant pathology, whereas Gleason 7 or greater is thought to be significant disease, irrespective of volume. As such, volume of disease adds very little to the decision-making process.

Currently, the proposed framework has been implemented as a research tool, and once more evaluations are conducted, the tool will be developed as a simple to use application which can be made accessible to clinicians. The tool will take the clinical test results (i.e. age at diagnosis, PSA, biopsy Primary and Secondary Gleason patterns, and clinical T stage) of an individual patient and predict his likelihood of having extra-prostatic cancer, and thereby aid the clinical decision-making process. Ongoing work is applying the proposed neuro-fuzzy predictor to a larger dataset, examining other computational intelligence approaches, and continuing the development of novel algorithms for predicting disease status in patients with prostate cancer.
